# Polyion complex micelle formed from tetraphenylethene containing block copolymer

**DOI:** 10.1186/s40824-017-0103-9

**Published:** 2017-10-10

**Authors:** Seong Min Lee, Woo-Dong Jang

**Affiliations:** Department of Chemistry, Yonsei University, 50 Yonsei-ro, Seodaemun-gu, Seoul, 03722 Republic of Korea

**Keywords:** Aggregation induced emission, Polyion complex, Photodynamic therapy, Dendrimer, Polyoxazoline

## Abstract

**Background:**

Polymeric micelles attract great attention in drug delivery and therapeutics. Various types of block copolymers have been designed for the application in biomedical fields. If we can introduce additional functional groups to the block copolymers, we can achieve advanced applications. In this regards, we tried to introduce aggregation induced emission enhancement (AIE) unit in the block copolymer.

**Methods:**

The formation of polyion complex micelle was confirmed by dynamic light scattering and transmission electron microscopy. HeLa cells were incubated with polyion complex micelle and broad-band visible light using a halogen lamp (150 W) was irradiated to evaluate photocytotoxicity of polyion complex (PIC) micelle.

**Results:**

For the design of functional polymeric micelle, aggregation induced emission enhancement unit was introduced in the middle of block copolymer. We newly synthesized a new type block copolymer (PEG-TPE-PEI) possessing tetraphenylethene (TPE) group, as an AIE unit, in the middle of polymeric segments of PEG and PEI, which successfully formed PIC micelle with DP. The formation of PIC micelle was confirmed by dynamic light scattering, ζ potential measurement and transmission electron microscopy.

**Conclusions:**

PEG-TPE-PEI successfully formed PIC micelle by mixing with negatively charged dendrimer porphyrin. The PIC micelle exhibited photocytotoxicity upon illumination of broadband visible light.

## Background

Polymeric micelles consist of hydrophobic inner core and hydrophilic outer shell attract great attention in drug delivery and therapeutics [1–3]. Various interactions can be utilized for the formation of polymeric micelles. A block copolymer having hydrophilic and hydrophobic blocks can form stable polymeric micelle in aqueous media. The difference in the internal and external properties of the micelle enables hydrophobic drugs to more bioavailable when used in the body. A block copolymer having hydrophilic block and ionic block also can form stable polyion complex (PIC) micelle when it interact with oppositely charged polymers [4, 5]. For the formation of stable PIC micelle, block copolymers with poly(ethylene glycol) (PEG) and electrolytes such as poly(L-lysine) (PLL), poly(L-glutamate), and polyethyleneimine (PEI) have been conjugated. For example, PEG-b-PLL has been used for the formation of DNA- or dendrimer-containing PIC micelle [6–8]. Especially, negatively charged dendrimer porphyrin (DP)-containing PIC micelle was extensively studied for the application in photodynamic therapy [9–16].

Aggregation induced emission (AIE) is anomalous photophysical phenomenon that observed in several organic fluorophores [17–19]. Generally, organic fluorophores having planar structure show collisional quenching behaviors in high concentration. By the formation of aggregates, most organic fluorophores become non-fluorescent. However, some organic fluorophores having rotatable groups show enhanced fluorescence emission by restricted molecular motion at solid state. Tetraphenylethene (TPE) group would be a typical example of AIE moiety. The phenyl units in TPE can be freely rotate in solution state through propeller motion. Therefore, the fluorescence emission of TPE in solution state is very weak because the excitation energy is released through non-radiative decay pathway. In contrast, the aggregate of TPE sterically restrict molecular motion of the phenyl units. Therefore, the fluorescence emission of TPE can be increased by aggregation.

## Methods

### Materials and measurements

All commercially available reagents were reagent grade and used without further purification. CH_2_Cl_2_, hexane, and tetrahydrofuran (THF) were freshly distilled before each use. Recycling preparative SEC was performed on a LC-9201 (JAI, Tokyo, Japan) instrument equipped with JAIGEL-1H, JAIGEL-2H, and JAIGEL-3H columns using CHCl_3_ as the eluent. UV-Vis absorption spectra were measured using a V-660 spectrophotometer (JASCO, Tokyo, Japan) equipped with a thermostatic cell holder coupled with a controller (ETCS-761, JASCO, Tokyo, Japan) at 30 °C. Fluorescence spectra were measured by a JASCO FP-6300 spectrophotometer equipped with a thermostatic cell holder (ETC-273 T, JASCO, Tokyo, Japan) coupled with a controller (ETC-273 T, JASCO, Tokyo, Japan) at 30 °C. All fluorescence spectra were measured using under-400 nm-cut off filter over detector and excitation wavelength in all measurements is fixed at 365 nm. All spectral measurements were carried out using a quartz cuvette with a path length of 1 cm. ^1^H–NMR spectra were recorded using a Bruker DPX 400 (400 MHz) spectrometer in CD_2_Cl_2_. Analytical SEC was performed on a JASCO HPLC equipped with HF-403HQ and HF-404HQ columns (Shodex, Tokyo, Japan) using THF as the eluent. MALDI-TOF-MS was performed on a Bruker Daltonics LRF20 with dithranol as the matrix. The DLS measurements were performed using a Photal dynamic laser scattering DLS-7000 spectrometer (Otsuka Electronics Co., Ltd., Osaka, Japan) equipped with GLG3050 488 nm Ar laser (NEC Co., Ltd., Japan) and/or Zetasizer Nano ZS-90 (Malvern Co., Ltd., USA) with 532 nm laser irradiation.

### Synthesis

1. To a 500 mL two necked round bottom flask, phenylboronic acid (10.0 g, 82.0 mmol), Pd(OAc)_2_ (0.122 g, 0.5 mmol), triphenylphosphine (0.286 g, 0.001 mmol), α-chloro-p-xylene (7.17 mL, 0.041 mmol) and K_3_PO_4_ (23.18 g, 0.110 mmol) was added into 150 mL toluene under N_2_ atmosphere, and stirred for 20 h at 80 °C. Then, the reaction mixture was cooled to room temperature and washed with NaOH solution (1.00 M). The organic layer was purified by flash column chromatography on silica gel using hexane and dichloromethane to afford phenylmethane derivatives. 8.85 g (64%) as a yellow liquid. ^1^H NMR (400 MHz, CD_2_Cl_2_) *δ* = 7.66–7.15 (m, 9 H), 3.98 (s, 2 H), 2.361 (s, 3 H).
2. To a 4-methyl diphenylmethane (3.5 g, 19.2 mmol) solution in THF under N_2_, n-butyllithium (17 mL, 1.6 M in hexane, 27.2 mmol) was slowly added by syringe with stirring at 0 °C for 1 h. Then, 4-methylbenzophenone (5.00 g, 25.4 mmol) was slowly added, and the solution was further stirred for overnight at room temperature. The reaction mixture was quenched with saturated NH_4_Cl solution and then extracted with CH_2_Cl_2_. The organic layers are collected and concentrated. The crude product and p-toluenesulfonic acid (0.2 g) are dissolved into 100 mL of toluene. The mixture was heated to reflux for 4 h. After cooled down to room temperature, the reaction mixture was extracted with CH_2_Cl_2_. The combined organic layer was was purified by silica-gel chromatography using hexane as eluent to give **2** as white solid (1.99 g). ^1^H NMR (CD_2_Cl_2_, 400 MHz), *δ* = 7.52–6.97 (m, 18 H), 2.29–2.26 (t, 6 H).
3. To a CCl_4_ solution of **2** (0.70 g, 1.94 mmol) and *N*-bromosuccimide (0.70 g, 3.88 mmol), benzoyl peroxide (2 mg) was added and refluxed for 12 h. The mixture was extracted with CH_2_Cl_2_ and water. The combined organic layers was dried over magnesium sulfate, and evaporated under reduced pressure. The crude product was dissolved in *N*,*N*-dimethylformamide and sodium azaide (0.041 g) was added. And the mixture was stirred for 5 h at 50 °C. Then, the reaction mixture was extracted with CH_2_Cl_2_. The crude product was purified by silica-gel chromatography using hexane as eluent to give **3** as yellow solid (0.154 g, 18% yield). ^1^H NMR (CD_2_Cl_2_, 400 MHz), δ = 7.13–7.011 (m, 18 H), 4.27 (s, 4 H).

### PiPrOx

An acetonitrile solution (15 mL) of methyl p-toluenesulfonate (0.28 mL, 1.62 mmol) and iPrOx (10 mL, 73.2 mmol) was stirred at 40 °C under N_2_ atmosphere and monitored with analytical SEC and MALDI-TOF-MS. When the reaction was completed, the reaction mixture was cooled to room temperature and further stirred for 4 days after addition of N-methyl propargylamine (0.183 mL, 112 mmol) to introduce propargyl group at the ω-terminal. The solution of PiPrOx was purified via dialysis for 2 days against distilled water and then recovered by lyophilization to obtain **PiPrOx** as white powder (5.30 g, 90%). ^1^H–NMR (400 MHz, CD_2_Cl_2_, 25 °C) *δ* (ppm): 3.48 (broad s; −CH_2_-CH_2_- on the polymer backbone), 3.06 (s; terminal -CH_3_), 2.96–2.54 (two broad s; −CH- on the polymer side chain), 2.36–2.26 (two broad s; −NCH_2_- on the polymer side chain), 1.07 (strong broad s; −CH_3_ on the polymer side chain).

### TPE-PiPrOx

A mixture solution of **3** (160 mg, 0.032 mmol), PiPrOx (1.28 g, 0.40 mmol) and copper(II) sulfate pentahydrate (201.6 mg, 0.67 mmol) in 20 mL of THF were placed in round bottom flask. Aqueous solution (1 mL) of sodium ascorbate (713 mg, 0.67 mmol) was added and refluxed for 1 days. The mixture was washed with brine and water. The organic layer was concentrated in vacuo and purified with recycling preparative SEC and then recovered by lyophilization to obtain **TPE-PiPrOx** as yellow powder (123 mg). ^1^H–NMR (400 MHz, CD_2_Cl_2_, 25 °C) δ (ppm): 7.28 (broad s, 6 H; −CH on TPE), 7.1 (broad s, 12 H; −CH in TPE), 4.23 (broad s, 2 H; −CH_2_-N_3_), 3.48 (broad s; −CH_2_-CH_2_- on the polymer backbone), 3.06 (s; terminal -CH_3_), 2.96–2.54 (two broad s; −CH- on the polymer side chain), 2.36–2.26 (two broad s; −NCH_2_- on the polymer side chain), 1.07 (strong broad s; −CH_3_ on the polymer side chain).

### PEG-TPE-PiPrOx

To a mixture solution of **TPE-PiPrOx** (500 mg, 10.0 mmol), propargyl-bearing PEG (583 mg, 11.6 mmol), and copper(II) sulfate pentahydrate (63 mg, 35 mmol) in 5 mL of THF, aqueous solution (1.5 mL) of sodium ascorbate (223 mg) was added and refluxed for 1 days. Then, the reaction mixture was washed with brine and water. The combined organic layer was concentrated in vacuo and purified with recycling preparative SEC and then recovered by lyophilization to obtain **PEG-TPE-PiPrOx** as yellow powder (123 mg, 18%). ^1^H–NMR (400 MHz, CD_2_Cl_2_, 25 °C) δ (ppm): 7.4 (broad s, 10 H; −CH on TPE), 7.1 (broad s, 8 H; −CH in TPE), 3.69 (broad s; −CH_2_-CH_2_- on the PEG backbone), 3.43 (s; broad s; −CH_2_-CH_2_- on the iPrPOx backbone), 3.33 (broad s; terminal -CH_3_ on the PEG backbone), 3.06 (s; terminal -CH_3_), 2.96–2.54 (two broad s; −CH- on the iPrPOx side chain), 2.36–2.26 (two broad s; −NCH2- on the polymer side chain), 1.07 (strong broad s; −CH_3_ on the polymer side chain).**PEG-TPE-PEI**: **PEG-TPE-PiPrOx** (50 mg) was dissolved in 5.00 M of aqueous HCl (10.0 mL) and refluxed for 1 days. The 2.5 M NaOH solution was added to mixture until pH reached to 8.0. **PEG-TPE-PEI** was recovered by lyophilization as a red powder. ^1^H–NMR (400 MHz, CD_2_Cl_2_, 25 °C) δ (ppm): 7.27 (broad s, 10 H; −CH on TPE), 7.1 (broad s, 8 H; −CH in TPE), 3.69 (broad s; −CH_2_-CH_2_- on the PEG backbone), 3.43 (s; broad s; −CH_2_-CH_2_- on the PEI backbone), 3.33 (broad s; terminal -CH_3_ on the PEG backbone), 3.06 (s; terminal -CH_3_), 1.87–1.78 (two broad s; −NCH_2_- on the PEI side chain).

### Preparation of PIC micelle

PIC micelles were obtained from negatively charged DP and **PEG-TPE-PEI**. In a typical procedure, the **PEG-TPE-PEI** was dissolved in an aqueous NaH_2_PO_4_ solution and added to an aqueous solution of DP in PBS buffer to give a solution containing PIC micelles. The ratio of positive charge to negative charge was fixed at 1:1.

### Cytotoxicity assay

HeLa cells were used in the cell culture studies. In the cytotoxicity assay, different concentration of PEG-TPE-PEI, DP, and PIC micelle in Dulbecco’s modified Eagle’s medium (10% FBS, MEM) were added to cells in 96-wll culture plates (*n* = 4). After a 24 h incubation at 37 °C, cells were washed with PBS, and then plates were photoirradiated for 15–60 min with broad-band visible light using a halogen lamp (150 W) equipped with a filter passing light of 400–700 nm (fluence energy; 27–107 kJ/m^2^). The viability of the cells was evaluated using mitochondrial respiration via the 3-(4,5-dimethyl thiazole-2-yl)-2,5-diphenyltetrazolium bromide cleavage assay (MTT assay) following incubation for 48 h after photoirradiation or washing in the case of the dark toxicity investigation.

## Results

**Scheme 1:**
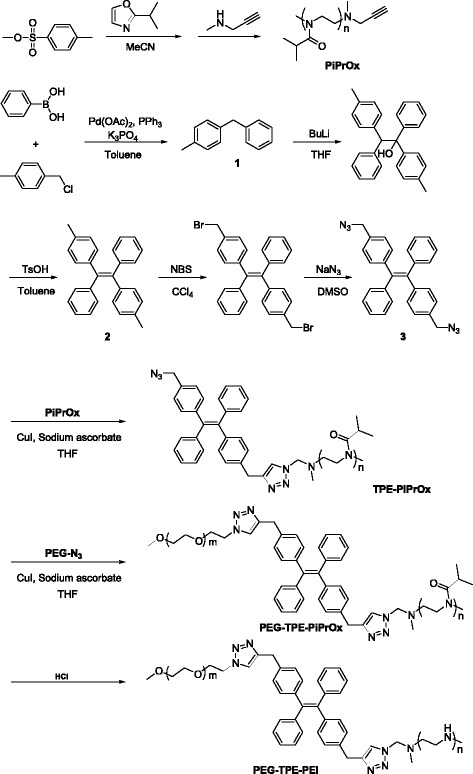
Synthesis of **PEG-TPE-PEI**

The preparation of **PEG-TPE-PEI** was summarized in Scheme 1. Briefly, poly(2-isopropyl-2-oxazoline) (**PiPrOx**) was prepared by living cationic polymerization of 2-isopropyl-2-oxazoline using methyl *p*-toluenesulfonate as an initiator [20]. The living end group of PiPrOx was terminated with *N*-methylpropargylamine to introduce clickable propargyl end group [21]. Then, phenylboronic acid and *p*-chloromethyl toluene was coupled to obtain **1**, which was further reacted with 4-methyl benzophenone and successive dehydration reaction was conducted to obtain **2**. Azide-bearing TPE (**3**) was obtained from **2** via bromination and azide coupling reactions. **PiPrOx** was introduced to **3** by copper catalyzed click reaction [22]. Then, propargyl-bearing PEG was again introduced to the other azide group in TPE. Finally, the PiPrOx chain was hydrolyzed by acid treatment to convert PEI block. After the reaction, preparative GPC was applied to purify the block copolymers. All the products were characterized by ^1^H NMR and size exclusion chromatography (SEC) measurements.

**Fig. 1:**
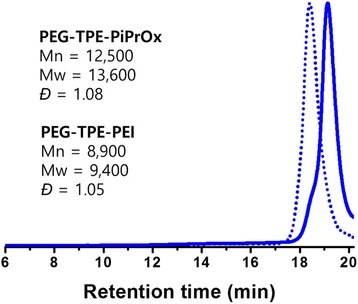
SEC profiles of **PEG-TPE-PiPrOx** and **PEG-TPE-PEI**

The number average molecular weight (*M*n) and dispersity index (*Đ*) of **PEG-TPE-PiPrOx** were about 12,500 g/mol and 1.08, respectively, indicating narrow molecular weight distribution. Likewise, PEG-TPE-PEI also has very narrow molecular weight distribution, where *M*n and *Đ* values were about 8900 g/mol and 1.05, respectively (Fig. 1). The elution time of **PEG-TPE-PEI** was longer than that of **PEG-TPE-PiPrOx**.

**Fig. 2:**
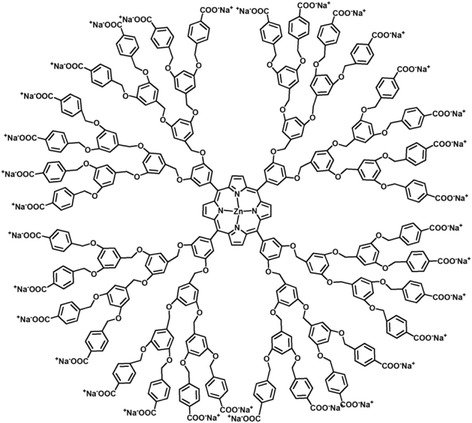
Chemical structure of **DP**

Anionic **DP** was prepared by previously reported procedure. The structure of **DP** is shown in Fig. 2. Because **PEG-TPE-PEI** has cationic polymer block, we have tested the formation of PIC micelle with the anionic **DP**. For the formation of PIC micelle, **PEG-TPE-PEI** (13.0 mg) and **DP** (7.3 mg) was dissolved in 10 mM phosphate buffered saline (PBS, pH 7.4) in a stoichiometric ratio of positive and negative charges, respectively. The formation of PIC micelle was confirmed by ζ-potential and dynamic light scattering (DLS) measurements.

**Fig. 3:**
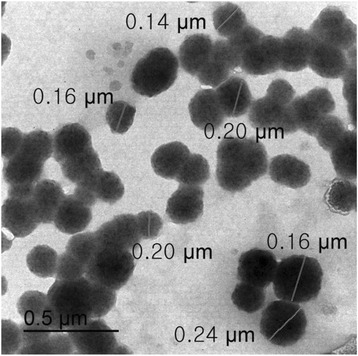
TEM image of PIC micelle

DLS measurement of **PEG-TPE-PEI** showed formation of nanoparticles with an average diameter of 119.2 nm. The nanoparticle can be formed by hydrophobic attraction of TPE units. On the other hand, when we mixed **PEG-TPE-PEI** with **DP** in a 1:1 charge ratio, uniform sized PIC micelle with an average diameter of 87 nm was formed. The ζ potential of **DP** was confirmed to be −16.87 mV. But the PIC micelles showed −4.12 mV of surface potential. The reduced ζ potential values indicates the surface of PIC micelle covered by PEG segments. The formation of PIC micelle was again confirmed by transmission electron microscopy (TEM) measurement. As shown in Fig. 3, TEM image of PIC micelles (1.5 mg/mL) showed spherical shapes with approximately 100–200 nm sizes.

**Fig. 4:**
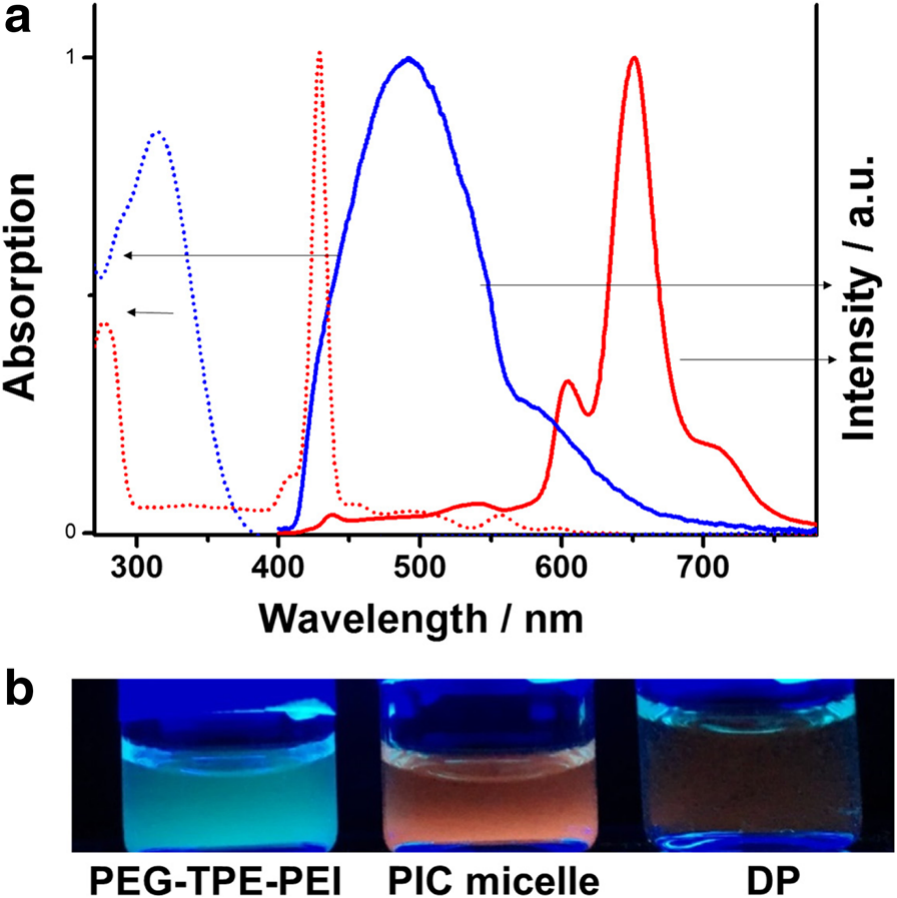
**a**) Absorption (dotted line) and emission (solid line) spectra of **DP** (red line) and **3** (blue line), **b**) fluorescence emission of **PEG-TPE-PEI**, PIC micelle, and **DP**

Figure 4a shows absorption and emission spectra of **3** and **DP**. Because the emission of **3** is well overlaps with the Soret absorption band of **DP**, we can expect efficient energy transfer from TPE to **DP**. As expected, the fluorescence of PIC micelle exhibited stronger fluorescence emission than that of **DP** alone, indicating the efficient energy transfer.

**Fig. 5:**
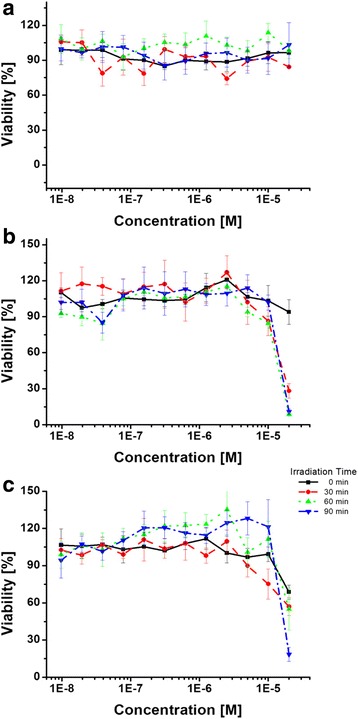
Photocytotoxicity of **a**) **PEG-TPE-PEI**, **b**) **DP**, and **c**) PIC micelles

Because **DP** is an effective photosensitizer for photodynamic therapy (PDT), we have measured photocytotoxicity of the PIC micelle. The PDT efficacy was confirmed by cell viability measurement using MTT assay for various light irradiation time and concentration. HeLa cells (3000 cells/well) were incubated with PIC micelle and **DP** for 24 h. A broad-band visible light from a light emitting diode (LED; incident energy 132 kJcm^−2^) was illuminated to the cells for 0, 30, 60, and 90 min, respectively. Under dark conditions, the cell viability for both **DP** and PIC micelle containing well were almost negligible, whereas strong cytotoxicity was observed for both **DP** and PIC micelle containing well by increasing light exposure time (Fig. 5).

## Discussion

We newly synthesized a new type block copolymer (**PEG-TPE-PEI**) possessing TPE group as an AIE unit in the middle of polymeric segments of PEG and PEI, which successfully formed PIC micelle with DP [23–26]. In general, fluorescent dyes show collisional quenching behaviors highly concentrated state. Therefore, most fluorescent dyes become non-fluorescent when they are encapsulated into the micellar formulation. However, TPE groups can show enhanced fluorescence emission by the formation of polymeric micelle. Such aspect will give great advantages for the monitoring of micelles [17–19].

Through living cationic polymerization, **PiPrOx** was successfully synthesized. **PiPrOx** and PEG were introduced to the azide-bearing TPE by copper catalyzed click reaction. **PiPrOx** chain was hydrolyzed by acid treatment to convert PEI block. All the products were characterized by ^1^H NMR and SEC measurements. The results of ^1^H NMR and SEC measurement indicated narrow molecular weight distribution of **PEG-TPE-PiPrOx** and **PEG**-**TPE**-**PEI**.

The formation of PIC micelle was confirmed by ζ-potential and dynamic light scattering (DLS) measurements. The result of DLS measurement indicated the formation of uniform sized PIC micelle. The change of ζ potential also indicated the successful formation of PIC micelles. TEM observation further evidenced the formation of uniform PIC micelles.

Because the absorption band of **DP** overlaps with the emission of TPE unit, PIC micelle exhibited enhanced fluorescence emission than that of **DP** alone due to the energy transfer from TPE units to **DP**s. The **DP**-encapsulated PIC micelle exhibited strong cytotoxicity under broad-band visible light illumination. The PIC micelle exhibited almost comparable photocytotoxicity with **DP**. Because **PEG**-**TPE**-**PEI** successfully formed PIC micelle with **DP**, other anionic macromolecules can be encapsulated by electrostatic interaction with positively charged **PEI** block in **PEG**-**TPE**-**PEI**.

## Conclusion

We have prepared a new type block copolymer (**PEG-TPE-PEI**) possessing TPE group as an AIE unit in the middle of polymeric segments of PEG and PEI, which successfully formed PIC micelle with **DP**. The formation of PIC micelle was confirmed by dynamic light scattering, ζ potential measurement and transmission electron microscopy. The PIC micelle exhibited photocytotoxicity upon illumination of broadband visible light. Because the TPE group can emit enhanced fluorescence, the formation of polymeric micelle can be directly observed. If we use non-fluorescent drug to form polymeric micelle, the AIE phenomena can be greatly useful.

## Abbreviation

AIE: Aggregation induced fluorescence enhancement
DLS: Dynamic light scattering
DP: Dendrimer porphyrin
LED: Light emitting diode
PDT: Photodynamic therapy
PEG: Poly(ehtyleneglycol
PEI: Polyethyleneimine
PIC: Polyion complex
PiPrOx: Poly(2-isopropyl-2-oxazoline)
TEM: Transmission electron microscopy
THF: Tetrahydrofuran
TPE: Tetraphenylethane
